# 4,5-Bis(2,4-di-*tert*-butyl­phen­oxy)phthalonitrile

**DOI:** 10.1107/S1600536811006118

**Published:** 2011-02-26

**Authors:** Johannes H. van Tonder, Theunis J. Muller, Barend C. B. Bezuidenhoudt

**Affiliations:** a Department of Chemistry, University of the Free State, PO Box 339, Bloemfontein 9300, South Africa

## Abstract

In the title compound, C_36_H_44_N_2_O_2_, the dihedral angles between the phthalonitrile ring and the two di-*tert*-butyl­benzene rings are 68.134 (8) and 70.637 (11)°. The two nitrile groups are almost coplanar with the phthalonitrile ring except for one of the N atoms which deviates from the plane by 0.125 (4) Å. One of the *tert*-butyl groups is disordered over two orientations, with refined occupancies of 0.814 (6) and 0.186 (6). Intra­molecular C—H⋯O inter­actions stabilize the molecular structure. The crystal packing is stabilized by inter­molecular C—H⋯N inter­actions.

## Related literature

For similar structures, see: Kartal *et al.* (2006 ▶); Petek *et al.* (2004 ▶); Dinçer *et al.* (2004 ▶). For other related structures, see: Şahin, *et al.* (2007 ▶); Wu *et al.* (2010 ▶); Yazıcı *et al.* (2004 ▶). For general background to phthalocyanines and metallophthalocyanines, see: Lenznoff & Lever (1989–1996 ▶); McKeown (1998 ▶); Wöhrle (2001 ▶).
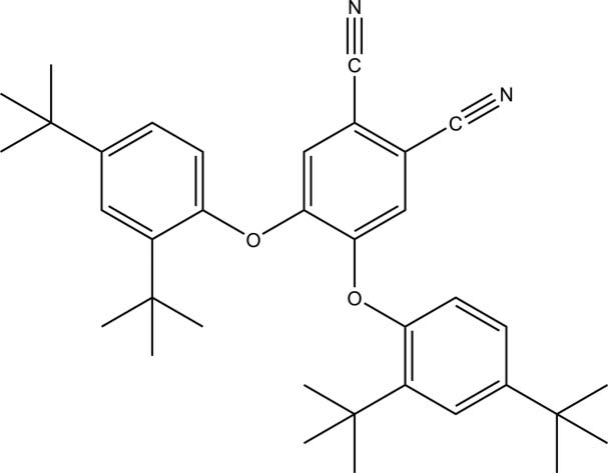


## Experimental

### 

#### Crystal data

C_36_H_44_N_2_O_2_
*M* *_r_* = 536.76
Triclinic, 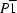

*a* = 10.9468 (3) Å
*b* = 11.0416 (4) Å
*c* = 15.3133 (5) Å
α = 99.719 (1)°
β = 102.996 (1)°
γ = 110.963 (1)°
*V* = 1619.71 (9) Å^3^
*Z* = 2
Mo *K*α radiation
μ = 0.07 mm^−1^
*T* = 175 K
0.21 × 0.19 × 0.14 mm

#### Data collection

Bruker APEXII CCD diffractometer
Absorption correction: multi-scan (*SADABS*; Bruker, 2008 ▶) *T*_min_ = 0.986, *T*_max_ = 0.990
31007 measured reflections
7785 independent reflections
5255 reflections with *I* > 2σ(*I*)
*R*_int_ = 0.031

#### Refinement

*R*[*F* ^2^ > 2σ(*F* ^2^)] = 0.052
*wR*(*F* ^2^) = 0.148
*S* = 1.03
7785 reflections
399 parameters
3 restraints
H-atom parameters constrained
Δρ_max_ = 0.29 e Å^−3^
Δρ_min_ = −0.30 e Å^−3^

### 

Data collection: *APEX2* (Bruker, 2008 ▶); cell refinement: *SAINT-Plus* (Bruker, 2008 ▶); data reduction: *SAINT-Plus* and *XPREP* (Bruker, 2008 ▶); program(s) used to solve structure: *SHELXS97* (Sheldrick, 2008 ▶); program(s) used to refine structure: *SHELXL97* (Sheldrick, 2008 ▶); molecular graphics: *DIAMOND* (Brandenberg & Putz, 2005 ▶); software used to prepare material for publication: *WinGX* (Farrugia, 1999 ▶).

## Supplementary Material

Crystal structure: contains datablocks global, I. DOI: 10.1107/S1600536811006118/lr2003sup1.cif

Structure factors: contains datablocks I. DOI: 10.1107/S1600536811006118/lr2003Isup2.hkl

Additional supplementary materials:  crystallographic information; 3D view; checkCIF report

## Figures and Tables

**Table 1:** Hydrogen-bond geometry (Å, °)

*D*—H⋯*A*	*D*—H	H⋯*A*	*D*⋯*A*	*D*—H⋯*A*
C19—H19*B*⋯O1	0.96	2.5	3.117 (2)	122
C20—H20*A*⋯O1	0.96	2.32	2.982 (3)	125
C36—H36*B*⋯O2	0.96	2.52	3.122 (3)	121
C37—H37*B*⋯O2	0.96	2.29	2.966 (2)	127
C22*A*—H22*A*⋯N2^i^	0.96	2.59	3.535 (4)	170

Symmetry code: (i) 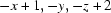.

**Fig. 1.**
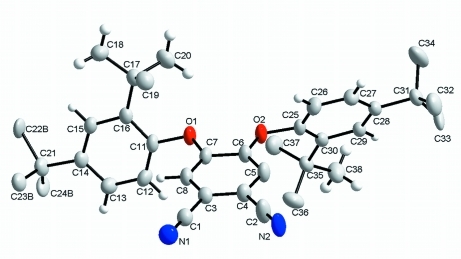
Diamond representation of the title compound, showing the numbering scheme and displacement ellipsoids (50% probability). Some H atoms and the disorder was left out for clarity.

## supplementary crystallographic information

### Comment

Substituted phthalonitriles have been used as starting materials for synthesizing peripherally substituted phtalocyanines and subphthalocyanines (McKeown, 1998). Phthalocyanines and metallophthalocyanines have been invesitigated for many years because of their wide range of applications, including use in chemical sensors, liquid crystals, Langmiur-Blodgett films, non-linear optics, batteries, and as carrier generation materials in the near-infrared (Lennoff & Lever, 1989–1996). Some phthalocyanines have been used in the petroleum industry as catalysts for the oxidation of sulfur compounds in the gasoline fraction. Applications such as photoconducters in the xerographic double layers of laser printers and coping machines, and as as active materials in writable data-storage disks, are also known. The production of phthalocyanines for use in dyes and pigments is around 80 000 tonnes per year (Wöhrle, 2001). The crystal structure of the title compound is presented here. It containes three aromatic rings. Ring A (C3—C8, r.m.s = 0.0047), ring B (C11—C16, r.m.s = 0.0051) and ring C (C25—C30, r.m.s = 0.0038) are essentialy planar. C1, C2 and N1 is coplanar to ring A but N2 is -0.1252 (41) Å out of the plane formed by ring A. The C1≡N1 and the C2≡N2 triple bond distances are 1.145 (2) Å and 1.143 (2) Å respectively and are consistent with values found in similar compounds (Kartal *et al.* 2006, Petek *et al.* 2004 and Dinçer *et al.* 2004). The N1—C1—C3 and N2—C2—C4 bond angles are 179.26 (18) ° and 178.4 (3) ° respectively, this is consistent with values found for simular compounds (Şahin, *et al.* 2007, Wu, *et al.* 2010 and Yazıcı, *et al.* 2004). The dihedral angles between rings A and B and between rings A and C are 68.134 (8) ° and 70.637 (11) ° respectively. The angle between rings B and C is 48.12 (6) °. The crystal packing is stabilized by C—H···O intermolecular hydrogen interactions.

### Experimental

Ground K_2_CO_3_ (4.91 g; 35.5 mmol; 7 eq.) was added to a solution of 4,5-dichlorophthalonitrile (1.00 g; 5.08 mmol) and 2,4-di-*tert*-butylphenol (2.20 g; 10.7 mmol; 2.1 eq.) in dry DMF (75 ml) before stirring overnight at 80 °C. The reaction mixture was cooled to room temperature before being transferred to 3*M* HCl (80 ml conc. HCl in 200 ml H_2_O). The precipitate was filtered off, washed with H_2_O and allowed to dry in air. The crude product was recrystallized from hot ethyl acetate and ethanol (1:1) to yield the title compound (77.9%). *R_f_* 0.8 (Hexane:Acetone; 8:2); Mp 269.0 °C;

^1^H NMR (600 MHz, CDCl_3_) δ 7.52 (2*H*, d, *J* = 2.3 Hz, H-3', 3"), 7.31 (2*H*, dd, *J* = 8.4, 2.3 Hz, H-5', 5"), 7.21 (2*H*, s, H-3,6), 6.86 (2*H*, d, *J* = 8.4 Hz, H-6', 6" H-2,6), 1.39 (36*H*, s, –C(CH_3_)_3_). ^13^C NMR (151 MHz, CDCl_3_) δ 152.51, 150.60, 148.46, 140.82, 125.13 (C-3',3"), 124.74 (C-5',5"), 121.66 (C-3,6), 120.36 (C-6',6"), 115.42 (–CN), 109.64 (C-1,2), 35.03 (–C(CH_3_)_3_), 34.82 (–C(CH_3_)_3_), 31.57 (–C(CH_3_)_3_), 30.40 (–C(CH_3_)_3_).

### Refinement

The aromatic H atoms were placed in geometrically idealized positions and constrained to ride on its parent atoms with *U*_iso_ (H) = 1.2*U*_eq_(C) and at a distance of 0.93 Å. The methyl H atoms were placed in geometrically idealized positions and constrained to ride on its parent atoms with *U*_iso_(H) = 1.5*U*_eq_(C) and at a distance of 0.96 Å.

### Crystal data

**Table:**

C_36_H_44_N_2_O_2_	*Z* = 2
*M**_r_* = 536.76	*F*(000) = 580
Triclinic, *P*1	*D*_x_ = 1.1 Mg m^−^^3^
*a* = 10.9468 (3) Å	Mo *K*α radiation, λ = 0.71073 Å
*b* = 11.0416 (4) Å	Cell parameters from 7569 reflections
*c* = 15.3133 (5) Å	θ = 2.8–28.6°
α = 99.719 (1)°	µ = 0.07 mm^−^^1^
β = 102.996 (1)°	*T* = 175 K
γ = 110.963 (1)°	Cuboid, colourless
*V* = 1619.71 (9) Å^3^	0.21 × 0.19 × 0.14 mm

### Data collection

**Table:**

Bruker APEXII CCD diffractometer	5255 reflections with *I* > 2σ(*I*)
graphite	*R*_int_ = 0.031
φ and ω scans	θ_max_ = 28°, θ_min_ = 3.3°
Absorption correction: multi-scan (*SADABS*; Bruker, 2008)	*h* = −14→14
*T*_min_ = 0.986, *T*_max_ = 0.990	*k* = −14→14
31007 measured reflections	*l* = −20→20
7785 independent reflections	

### Refinement

**Table:**

Refinement on *F*^2^	Primary atom site location: structure-invariant direct methods
Least-squares matrix: full	Secondary atom site location: difference Fourier map
*R*[*F*^2^ > 2σ(*F*^2^)] = 0.052	Hydrogen site location: inferred from neighbouring sites
*wR*(*F*^2^) = 0.148	H-atom parameters constrained
*S* = 1.03	*w* = 1/[σ^2^(*F*_o_^2^) + (0.0592*P*)^2^ + 0.5205*P*] where *P* = (*F*_o_^2^ + 2*F*_c_^2^)/3
7785 reflections	(Δ/σ)_max_ = 0.017
399 parameters	Δρ_max_ = 0.29 e Å^−^^3^
3 restraints	Δρ_min_ = −0.30 e Å^−^^3^

### Special details

**Table:**

Experimental. The intensity data was collected on a Bruker X8 ApexII 4 K Kappa CCD diffractometer using an exposure time of 40 s/frame. A total of 2019 frames were collected with a frame width of 0.5° covering up to θ = 28.57° with 99.4% completeness accomplished.
Geometry. All s.u.'s (except the s.u. in the dihedral angle between two l.s. planes) are estimated using the full covariance matrix. The cell s.u.'s are taken into account individually in the estimation of s.u.'s in distances, angles and torsion angles; correlations between s.u.'s in cell parameters are only used when they are defined by crystal symmetry. An approximate (isotropic) treatment of cell s.u.'s is used for estimating s.u.'s involving l.s. planes.
Refinement. Refinement of *F*^2^ against ALL reflections. The weighted *R*-factor *wR* and goodness of fit *S* are based on *F*^2^, conventional *R*-factors *R* are based on *F*, with *F* set to zero for negative *F*^2^. The threshold expression of *F*^2^ > 2σ(*F*^2^) is used only for calculating *R*-factors(gt) *etc*. and is not relevant to the choice of reflections for refinement. *R*-factors based on *F*^2^ are statistically about twice as large as those based on *F*, and *R*- factors based on ALL data will be even larger.

### Fractional atomic coordinates and isotropic or equivalent isotropic displacement parameters (Å^2^)

**Table:**

	*x*	*y*	*z*	*U*_iso_*/*U*_eq_	Occ. (<1)
C1	0.32485 (17)	−0.09576 (17)	0.99913 (12)	0.0399 (4)	
C2	0.2111 (2)	0.0801 (2)	1.06591 (15)	0.0601 (6)	
C3	0.27916 (16)	−0.02106 (15)	0.94090 (12)	0.0350 (3)	
C4	0.22520 (18)	0.06741 (17)	0.97435 (12)	0.0399 (4)	
C5	0.18274 (18)	0.14119 (17)	0.91845 (12)	0.0428 (4)	
H5	0.1479	0.201	0.9412	0.051*	
C6	0.19232 (16)	0.12561 (15)	0.82952 (11)	0.0344 (3)	
C7	0.24483 (15)	0.03433 (15)	0.79532 (11)	0.0325 (3)	
C8	0.28764 (16)	−0.03739 (15)	0.85123 (12)	0.0356 (4)	
H8	0.3225	−0.0972	0.8286	0.043*	
C11	0.32695 (16)	−0.03783 (15)	0.67397 (11)	0.0341 (3)	
C12	0.46705 (18)	0.02362 (17)	0.71765 (12)	0.0422 (4)	
H12	0.5042	0.099	0.7687	0.051*	
C13	0.55247 (17)	−0.02689 (18)	0.68553 (12)	0.0421 (4)	
H13	0.6467	0.0138	0.716	0.05*	
C14	0.49885 (16)	−0.13740 (16)	0.60855 (11)	0.0342 (3)	
C15	0.35667 (16)	−0.19584 (16)	0.56661 (11)	0.0335 (3)	
H15	0.32	−0.2701	0.5148	0.04*	
C16	0.26543 (16)	−0.15055 (15)	0.59701 (11)	0.0317 (3)	
C17	0.10949 (16)	−0.22154 (16)	0.54796 (12)	0.0364 (4)	
C18	0.07343 (19)	−0.33885 (19)	0.46378 (13)	0.0503 (5)	
H18A	0.1151	−0.3052	0.419	0.075*	
H18B	−0.0247	−0.3829	0.4359	0.075*	
H18C	0.1071	−0.4021	0.4836	0.075*	
C19	0.03676 (18)	−0.27803 (18)	0.61657 (13)	0.0458 (4)	
H19A	−0.0601	−0.327	0.585	0.069*	
H19B	0.0516	−0.205	0.6675	0.069*	
H19C	0.0734	−0.3373	0.64	0.069*	
C20	0.05301 (19)	−0.1242 (2)	0.51307 (14)	0.0495 (5)	
H20A	0.0705	−0.0513	0.5649	0.074*	
H20B	−0.0443	−0.1713	0.4826	0.074*	
H20C	0.0974	−0.0891	0.4698	0.074*	
C21	0.58891 (17)	−0.19560 (18)	0.56883 (12)	0.0414 (4)	
C25	0.09512 (16)	0.28072 (16)	0.79279 (11)	0.0334 (3)	
C26	−0.03540 (17)	0.22322 (16)	0.80045 (12)	0.0406 (4)	
H26	−0.0763	0.1312	0.7942	0.049*	
C27	−0.10495 (16)	0.30261 (16)	0.81741 (12)	0.0385 (4)	
H27	−0.1921	0.2638	0.8236	0.046*	
C28	−0.04634 (15)	0.43969 (15)	0.82533 (11)	0.0310 (3)	
C29	0.08501 (15)	0.49289 (15)	0.81665 (10)	0.0300 (3)	
H29	0.1251	0.5846	0.8218	0.036*	
C30	0.16077 (15)	0.41736 (15)	0.80073 (10)	0.0294 (3)	
C31	−0.12629 (16)	0.52644 (17)	0.84061 (12)	0.0371 (4)	
C32	−0.1880 (2)	0.5004 (2)	0.91925 (15)	0.0562 (5)	
H32A	−0.2375	0.5556	0.9278	0.084*	
H32B	−0.1156	0.5223	0.9759	0.084*	
H32C	−0.2498	0.4071	0.9035	0.084*	
C33	−0.0344 (2)	0.6768 (2)	0.8677 (2)	0.0792 (8)	
H33A	0.0028	0.698	0.8184	0.119*	
H33B	0.0394	0.6995	0.9237	0.119*	
H33C	−0.0876	0.7274	0.8781	0.119*	
C34	−0.2414 (3)	0.4900 (3)	0.75103 (16)	0.0823 (8)	
H34A	−0.203	0.5063	0.7014	0.124*	
H34B	−0.2919	0.5442	0.7592	0.124*	
H34C	−0.3022	0.3964	0.7358	0.124*	
C35	0.30789 (15)	0.48317 (16)	0.79499 (11)	0.0352 (4)	
C36	0.40825 (18)	0.4698 (2)	0.87618 (14)	0.0540 (5)	
H36A	0.5008	0.517	0.876	0.081*	
H36B	0.389	0.3762	0.8696	0.081*	
H36C	0.398	0.5077	0.9339	0.081*	
C37	0.31886 (19)	0.4175 (2)	0.70201 (13)	0.0485 (4)	
H37A	0.2603	0.4318	0.6517	0.073*	
H37B	0.2908	0.3224	0.6945	0.073*	
H37C	0.4124	0.4571	0.7017	0.073*	
C38	0.35244 (18)	0.63396 (18)	0.80263 (15)	0.0504 (5)	
H38A	0.29	0.6458	0.7532	0.076*	
H38B	0.4437	0.6712	0.7979	0.076*	
H38C	0.3515	0.6793	0.8616	0.076*	
N1	0.36199 (18)	−0.15552 (17)	1.04487 (12)	0.0546 (4)	
N2	0.1971 (3)	0.0875 (3)	1.13809 (15)	0.0937 (8)	
O1	0.24505 (12)	0.02227 (11)	0.70557 (8)	0.0384 (3)	
O2	0.15855 (12)	0.19450 (11)	0.76953 (8)	0.0400 (3)	
C22A	0.7447 (3)	−0.1104 (4)	0.6219 (2)	0.0569 (8)	0.814 (6)
H22A	0.7633	−0.1136	0.6856	0.085*	0.814 (6)
H22B	0.7686	−0.0186	0.6198	0.085*	0.814 (6)
H22C	0.7982	−0.1465	0.593	0.085*	0.814 (6)
C23A	0.5683 (3)	−0.1892 (5)	0.46811 (18)	0.0696 (12)	0.814 (6)
H23A	0.6292	−0.2192	0.4438	0.104*	0.814 (6)
H23B	0.5879	−0.0981	0.4658	0.104*	0.814 (6)
H23C	0.4748	−0.2464	0.4314	0.104*	0.814 (6)
C24A	0.5532 (5)	−0.3364 (4)	0.5778 (5)	0.0921 (16)	0.814 (6)
H24A	0.6091	−0.3733	0.5526	0.138*	0.814 (6)
H24B	0.4579	−0.3913	0.5441	0.138*	0.814 (6)
H24C	0.5696	−0.3348	0.6423	0.138*	0.814 (6)
C22B	0.5042 (14)	−0.2940 (15)	0.4618 (7)	0.050 (3)	0.186 (6)
H22D	0.5663	−0.3179	0.4347	0.075*	0.186 (6)
H22E	0.4646	−0.2471	0.425	0.075*	0.186 (6)
H22F	0.4323	−0.3743	0.4633	0.075*	0.186 (6)
C23B	0.6083 (13)	−0.2968 (14)	0.6183 (9)	0.0414 (4)	0.186 (6)
H23D	0.664	−0.3346	0.5936	0.062*	0.186 (6)
H23E	0.5203	−0.3674	0.6093	0.062*	0.186 (6)
H23F	0.653	−0.2532	0.6837	0.062*	0.186 (6)
C24B	0.7055 (16)	−0.0922 (14)	0.5672 (16)	0.075 (5)	0.186 (6)
H24D	0.7568	−0.0353	0.6294	0.112*	0.186 (6)
H24E	0.6791	−0.04	0.5286	0.112*	0.186 (6)
H24F	0.7615	−0.1295	0.5424	0.112*	0.186 (6)

### Atomic displacement parameters (Å^2^)

**Table:**

	*U*^11^	*U*^22^	*U*^33^	*U*^12^	*U*^13^	*U*^23^
C1	0.0435 (9)	0.0387 (9)	0.0422 (10)	0.0193 (8)	0.0179 (8)	0.0111 (8)
C2	0.0913 (16)	0.0783 (14)	0.0484 (12)	0.0639 (13)	0.0374 (11)	0.0255 (11)
C3	0.0367 (8)	0.0329 (8)	0.0402 (9)	0.0163 (7)	0.0168 (7)	0.0114 (7)
C4	0.0487 (9)	0.0455 (9)	0.0374 (10)	0.0275 (8)	0.0209 (8)	0.0120 (8)
C5	0.0564 (10)	0.0455 (10)	0.0435 (10)	0.0343 (9)	0.0254 (8)	0.0112 (8)
C6	0.0411 (8)	0.0319 (8)	0.0388 (9)	0.0208 (7)	0.0184 (7)	0.0099 (7)
C7	0.0371 (8)	0.0290 (7)	0.0362 (9)	0.0164 (6)	0.0180 (7)	0.0059 (6)
C8	0.0411 (8)	0.0318 (8)	0.0425 (10)	0.0208 (7)	0.0202 (7)	0.0091 (7)
C11	0.0445 (9)	0.0340 (8)	0.0359 (9)	0.0234 (7)	0.0224 (7)	0.0100 (7)
C12	0.0461 (9)	0.0396 (9)	0.0378 (10)	0.0166 (8)	0.0173 (8)	−0.0005 (7)
C13	0.0366 (8)	0.0496 (10)	0.0378 (10)	0.0167 (8)	0.0149 (7)	0.0042 (8)
C14	0.0397 (8)	0.0394 (9)	0.0332 (9)	0.0209 (7)	0.0200 (7)	0.0116 (7)
C15	0.0418 (8)	0.0324 (8)	0.0318 (8)	0.0191 (7)	0.0166 (7)	0.0069 (7)
C16	0.0383 (8)	0.0310 (8)	0.0348 (9)	0.0189 (6)	0.0176 (7)	0.0126 (7)
C17	0.0384 (8)	0.0378 (8)	0.0398 (9)	0.0203 (7)	0.0157 (7)	0.0125 (7)
C18	0.0424 (10)	0.0524 (11)	0.0477 (11)	0.0187 (8)	0.0093 (8)	0.0010 (9)
C19	0.0422 (9)	0.0455 (10)	0.0548 (12)	0.0171 (8)	0.0226 (8)	0.0187 (9)
C20	0.0474 (10)	0.0566 (11)	0.0579 (12)	0.0305 (9)	0.0182 (9)	0.0262 (10)
C21	0.0431 (9)	0.0505 (10)	0.0425 (10)	0.0268 (8)	0.0239 (8)	0.0111 (8)
C25	0.0409 (8)	0.0361 (8)	0.0316 (8)	0.0247 (7)	0.0142 (7)	0.0061 (7)
C26	0.0433 (9)	0.0299 (8)	0.0481 (11)	0.0149 (7)	0.0171 (8)	0.0061 (7)
C27	0.0319 (8)	0.0369 (9)	0.0446 (10)	0.0131 (7)	0.0140 (7)	0.0056 (7)
C28	0.0316 (7)	0.0362 (8)	0.0296 (8)	0.0189 (6)	0.0108 (6)	0.0069 (6)
C29	0.0320 (7)	0.0314 (8)	0.0306 (8)	0.0167 (6)	0.0113 (6)	0.0078 (6)
C30	0.0322 (7)	0.0365 (8)	0.0242 (8)	0.0192 (6)	0.0097 (6)	0.0071 (6)
C31	0.0359 (8)	0.0433 (9)	0.0433 (10)	0.0252 (7)	0.0173 (7)	0.0132 (8)
C32	0.0628 (12)	0.0676 (13)	0.0641 (14)	0.0428 (11)	0.0377 (11)	0.0227 (11)
C33	0.0718 (14)	0.0479 (12)	0.150 (3)	0.0396 (11)	0.0676 (16)	0.0295 (14)
C34	0.0867 (17)	0.133 (2)	0.0566 (15)	0.0862 (18)	0.0123 (12)	0.0214 (15)
C35	0.0315 (7)	0.0435 (9)	0.0369 (9)	0.0209 (7)	0.0142 (7)	0.0097 (7)
C36	0.0365 (9)	0.0752 (14)	0.0522 (12)	0.0262 (9)	0.0092 (8)	0.0212 (10)
C37	0.0474 (10)	0.0597 (12)	0.0464 (11)	0.0251 (9)	0.0264 (9)	0.0115 (9)
C38	0.0397 (9)	0.0461 (10)	0.0706 (14)	0.0170 (8)	0.0276 (9)	0.0161 (10)
N1	0.0664 (11)	0.0530 (10)	0.0526 (10)	0.0305 (8)	0.0185 (8)	0.0214 (8)
N2	0.160 (2)	0.137 (2)	0.0594 (13)	0.1165 (19)	0.0640 (14)	0.0490 (13)
O1	0.0530 (7)	0.0419 (6)	0.0368 (7)	0.0314 (6)	0.0243 (5)	0.0116 (5)
O2	0.0580 (7)	0.0415 (6)	0.0394 (7)	0.0352 (6)	0.0242 (6)	0.0129 (5)
C22A	0.0425 (14)	0.089 (2)	0.0473 (17)	0.0339 (14)	0.0231 (13)	0.0108 (15)
C23A	0.0579 (18)	0.117 (3)	0.0391 (15)	0.046 (2)	0.0224 (13)	0.0008 (17)
C24A	0.075 (2)	0.054 (2)	0.178 (5)	0.0412 (18)	0.072 (3)	0.033 (3)
C22B	0.066 (8)	0.061 (8)	0.037 (6)	0.044 (7)	0.023 (5)	0.000 (5)
C23B	0.0431 (9)	0.0505 (10)	0.0425 (10)	0.0268 (8)	0.0239 (8)	0.0111 (8)
C24B	0.059 (9)	0.071 (8)	0.105 (15)	0.029 (7)	0.055 (10)	0.007 (9)

### Geometric parameters (Å, °)

**Table:**

C1—N1	1.145 (2)	C27—C28	1.388 (2)
C1—C3	1.438 (2)	C27—H27	0.93
C2—N2	1.143 (3)	C28—C29	1.392 (2)
C2—C4	1.434 (3)	C28—C31	1.534 (2)
C3—C8	1.383 (2)	C29—C30	1.3999 (19)
C3—C4	1.395 (2)	C29—H29	0.93
C4—C5	1.393 (2)	C30—C35	1.542 (2)
C5—C6	1.376 (2)	C31—C34	1.518 (3)
C5—H5	0.93	C31—C33	1.525 (3)
C6—O2	1.3578 (19)	C31—C32	1.528 (2)
C6—C7	1.412 (2)	C32—H32A	0.96
C7—O1	1.3588 (19)	C32—H32B	0.96
C7—C8	1.376 (2)	C32—H32C	0.96
C8—H8	0.93	C33—H33A	0.96
C11—C12	1.379 (2)	C33—H33B	0.96
C11—C16	1.393 (2)	C33—H33C	0.96
C11—O1	1.4110 (17)	C34—H34A	0.96
C12—C13	1.384 (2)	C34—H34B	0.96
C12—H12	0.93	C34—H34C	0.96
C13—C14	1.383 (2)	C35—C36	1.529 (2)
C13—H13	0.93	C35—C38	1.533 (2)
C14—C15	1.394 (2)	C35—C37	1.536 (2)
C14—C21	1.532 (2)	C36—H36A	0.96
C15—C16	1.397 (2)	C36—H36B	0.96
C15—H15	0.93	C36—H36C	0.96
C16—C17	1.536 (2)	C37—H37A	0.96
C17—C18	1.529 (2)	C37—H37B	0.96
C17—C20	1.531 (2)	C37—H37C	0.96
C17—C19	1.539 (2)	C38—H38A	0.96
C18—H18A	0.96	C38—H38B	0.96
C18—H18B	0.96	C38—H38C	0.96
C18—H18C	0.96	C22A—H22A	0.96
C19—H19A	0.96	C22A—H22B	0.96
C19—H19B	0.96	C22A—H22C	0.96
C19—H19C	0.96	C23A—H23A	0.96
C20—H20A	0.96	C23A—H23B	0.96
C20—H20B	0.96	C23A—H23C	0.96
C20—H20C	0.96	C24A—H24A	0.96
C21—C24B	1.384 (13)	C24A—H24B	0.96
C21—C24A	1.500 (4)	C24A—H24C	0.96
C21—C23B	1.501 (12)	C22B—H22D	0.96
C21—C23A	1.525 (3)	C22B—H22E	0.96
C21—C22A	1.557 (3)	C22B—H22F	0.96
C21—C22B	1.650 (11)	C23B—H23D	0.96
C25—C26	1.382 (2)	C23B—H23E	0.96
C25—C30	1.390 (2)	C23B—H23F	0.96
C25—O2	1.4089 (17)	C24B—H24D	0.96
C26—C27	1.380 (2)	C24B—H24E	0.96
C26—H26	0.93	C24B—H24F	0.96
			
N1—C1—C3	179.26 (18)	C26—C27—C28	120.78 (15)
N2—C2—C4	178.4 (3)	C26—C27—H27	119.6
C8—C3—C4	119.70 (15)	C28—C27—H27	119.6
C8—C3—C1	120.12 (14)	C27—C28—C29	117.23 (13)
C4—C3—C1	120.18 (15)	C27—C28—C31	120.53 (13)
C5—C4—C3	120.20 (15)	C29—C28—C31	122.23 (14)
C5—C4—C2	120.12 (15)	C28—C29—C30	124.32 (14)
C3—C4—C2	119.67 (15)	C28—C29—H29	117.8
C6—C5—C4	119.99 (14)	C30—C29—H29	117.8
C6—C5—H5	120	C25—C30—C29	115.24 (13)
C4—C5—H5	120	C25—C30—C35	122.92 (13)
O2—C6—C5	125.57 (13)	C29—C30—C35	121.83 (14)
O2—C6—C7	114.72 (14)	C34—C31—C33	109.46 (19)
C5—C6—C7	119.68 (14)	C34—C31—C32	109.21 (17)
O1—C7—C8	124.80 (13)	C33—C31—C32	106.95 (16)
O1—C7—C6	115.18 (13)	C34—C31—C28	108.44 (14)
C8—C7—C6	120.00 (14)	C33—C31—C28	112.04 (14)
C7—C8—C3	120.42 (14)	C32—C31—C28	110.70 (13)
C7—C8—H8	119.8	C31—C32—H32A	109.5
C3—C8—H8	119.8	C31—C32—H32B	109.5
C12—C11—C16	122.65 (13)	H32A—C32—H32B	109.5
C12—C11—O1	117.89 (14)	C31—C32—H32C	109.5
C16—C11—O1	119.32 (14)	H32A—C32—H32C	109.5
C11—C12—C13	120.01 (16)	H32B—C32—H32C	109.5
C11—C12—H12	120	C31—C33—H33A	109.5
C13—C12—H12	120	C31—C33—H33B	109.5
C14—C13—C12	120.56 (16)	H33A—C33—H33B	109.5
C14—C13—H13	119.7	C31—C33—H33C	109.5
C12—C13—H13	119.7	H33A—C33—H33C	109.5
C13—C14—C15	117.32 (14)	H33B—C33—H33C	109.5
C13—C14—C21	122.73 (15)	C31—C34—H34A	109.5
C15—C14—C21	119.95 (14)	C31—C34—H34B	109.5
C14—C15—C16	124.54 (15)	H34A—C34—H34B	109.5
C14—C15—H15	117.7	C31—C34—H34C	109.5
C16—C15—H15	117.7	H34A—C34—H34C	109.5
C11—C16—C15	114.91 (14)	H34B—C34—H34C	109.5
C11—C16—C17	123.53 (13)	C36—C35—C38	107.50 (15)
C15—C16—C17	121.56 (14)	C36—C35—C37	110.04 (14)
C18—C17—C20	107.50 (15)	C38—C35—C37	107.12 (14)
C18—C17—C16	111.47 (13)	C36—C35—C30	109.04 (14)
C20—C17—C16	111.28 (14)	C38—C35—C30	111.76 (12)
C18—C17—C19	108.14 (15)	C37—C35—C30	111.30 (14)
C20—C17—C19	109.01 (14)	C35—C36—H36A	109.5
C16—C17—C19	109.35 (14)	C35—C36—H36B	109.5
C17—C18—H18A	109.5	H36A—C36—H36B	109.5
C17—C18—H18B	109.5	C35—C36—H36C	109.5
H18A—C18—H18B	109.5	H36A—C36—H36C	109.5
C17—C18—H18C	109.5	H36B—C36—H36C	109.5
H18A—C18—H18C	109.5	C35—C37—H37A	109.5
H18B—C18—H18C	109.5	C35—C37—H37B	109.5
C17—C19—H19A	109.5	H37A—C37—H37B	109.5
C17—C19—H19B	109.5	C35—C37—H37C	109.5
H19A—C19—H19B	109.5	H37A—C37—H37C	109.5
C17—C19—H19C	109.5	H37B—C37—H37C	109.5
H19A—C19—H19C	109.5	C35—C38—H38A	109.5
H19B—C19—H19C	109.5	C35—C38—H38B	109.5
C17—C20—H20A	109.5	H38A—C38—H38B	109.5
C17—C20—H20B	109.5	C35—C38—H38C	109.5
H20A—C20—H20B	109.5	H38A—C38—H38C	109.5
C17—C20—H20C	109.5	H38B—C38—H38C	109.5
H20A—C20—H20C	109.5	C7—O1—C11	118.38 (12)
H20B—C20—H20C	109.5	C6—O2—C25	120.00 (12)
C24B—C21—C24A	135.6 (7)	C21—C22A—H22A	109.5
C24B—C21—C23B	117.8 (9)	C21—C22A—H22B	109.5
C24A—C21—C23B	27.0 (4)	C21—C22A—H22C	109.5
C24B—C21—C23A	72.6 (9)	C21—C23A—H23A	109.5
C24A—C21—C23A	112.7 (3)	C21—C23A—H23B	109.5
C23B—C21—C23A	133.6 (5)	C21—C23A—H23C	109.5
C24B—C21—C14	110.0 (5)	C21—C24A—H24A	109.5
C24A—C21—C14	109.31 (17)	C21—C24A—H24B	109.5
C23B—C21—C14	108.7 (5)	C21—C24A—H24C	109.5
C23A—C21—C14	108.60 (16)	C21—C22B—H22D	109.5
C24B—C21—C22A	36.7 (9)	C21—C22B—H22E	109.5
C24A—C21—C22A	108.2 (2)	H22D—C22B—H22E	109.5
C23B—C21—C22A	84.1 (5)	C21—C22B—H22F	109.5
C23A—C21—C22A	106.40 (19)	H22D—C22B—H22F	109.5
C14—C21—C22A	111.68 (16)	H22E—C22B—H22F	109.5
C24B—C21—C22B	108.7 (8)	C21—C23B—H23D	109.5
C24A—C21—C22B	75.0 (5)	C21—C23B—H23E	109.5
C23B—C21—C22B	100.4 (6)	H23D—C23B—H23E	109.5
C23A—C21—C22B	40.0 (5)	C21—C23B—H23F	109.5
C14—C21—C22B	110.8 (4)	H23D—C23B—H23F	109.5
C22A—C21—C22B	133.0 (4)	H23E—C23B—H23F	109.5
C26—C25—C30	122.53 (13)	C21—C24B—H24D	109.5
C26—C25—O2	117.88 (14)	C21—C24B—H24E	109.5
C30—C25—O2	119.43 (13)	H24D—C24B—H24E	109.5
C27—C26—C25	119.90 (15)	C21—C24B—H24F	109.5
C27—C26—H26	120	H24D—C24B—H24F	109.5
C25—C26—H26	120	H24E—C24B—H24F	109.5

### Hydrogen-bond geometry (Å, °)

**Table:**

*D*—H···*A*	*D*—H	H···*A*	*D*···*A*	*D*—H···*A*
C19—H19B···O1	0.96	2.5	3.117 (2)	122
C20—H20A···O1	0.96	2.32	2.982 (3)	125
C36—H36B···O2	0.96	2.52	3.122 (3)	121
C37—H37B···O2	0.96	2.29	2.966 (2)	127
C22A—H22A···N2^i^	0.96	2.59	3.535 (4)	170

Symmetry codes: (i) −*x*+1, −*y*, −*z*+2.
